# DEMENTIA CARE PARTNER PREPAREDNESS AND DESIRE TO SEEK LONG-TERM CARE AT HOSPITAL DISCHARGE

**DOI:** 10.1093/geroni/igae098.1361

**Published:** 2024-12-31

**Authors:** Ashley Kuzmik

**Affiliations:** The Pennsylvania State University, University Park, Pennsylvania, United States

## Abstract

At hospital discharge, care partners of persons living with dementia encounter more complex caregiving demands and lack preparedness to manage the increased care needs, frequently resulting in the patient’s admission to long-term care. This study explored the mediating effects of patient clinical factors on the relationship between care partner preparedness and desire to seek long-term admission of persons with dementia at hospital discharge. The sample included 424 patient and care partner dyads from the Family centered Function-focused Care (Fam-FFC) trial. Using bootstrapping, we tested a multiple mediation model to examine the indirect effects of care partner preparedness on desire to seek long-term care admission through patient clinical factors (i.e., behavioral and psychological symptoms, comorbidities, delirium severity, physical function, and cognition). The results revealed that delirium severity (B=-.005; 95% bootstrap CI=-.015, -.001) and physical function (B=.004; 95% bootstrap CI=.002,.007) partially mediated the association between care partner preparedness and desire to seek long-term care admission. Findings highlight the importance of targeting delirium severity and physical function in clinical interventions for persons with dementia at hospital discharge to support care partner preparedness and minimize undesired long-term care admission.

